# Incidence, antimicrobial resistance and mortality of *Pseudomonas aeruginosa* bloodstream infections among hospitalized patients in China: a retrospective observational multicenter cohort study from 2017 to 2021

**DOI:** 10.3389/fpubh.2023.1294141

**Published:** 2024-01-05

**Authors:** Shuzhen Xiao, Xianghui Liang, Lizhong Han, Shengyuan Zhao

**Affiliations:** ^1^Department of Laboratory Medicine, Ruijin Hospital, Shanghai Jiao Tong University School of Medicine, Shanghai, China; ^2^Department of Clinical Microbiology, Ruijin Hospital, Shanghai Jiao Tong University School of Medicine, Shanghai, China; ^3^Department of Clinical Laboratory, Xiangya Hospital, Central South University, Changsha, Hunan, China

**Keywords:** *Pseudomonas aeruginosa*, bloodstream infections, incidence, mortality, antimicrobial resistance

## Abstract

**Background:**

*Pseudomonas aeruginosa (P. aeruginosa)* accounts for high antimicrobial resistance and mortality rates of bloodstream infections (BSIs). We aim to investigate incidence, antimicrobial resistance and risk factors for mortality of *P*. *aeruginosa* BSIs among inpatients.

**Methods:**

A retrospective cohort study were conducted at two tertiary hospitals in 2017–2021. Medical and laboratory records of all inpatients diagnosed with *P*. *aeruginosa* BSIs were reviewed. A generalized linear mixed model was used to identify risk factors for mortality.

**Results:**

A total of 285 patients with *P*. *aeruginosa* BSIs were identified. Incidence of *P*. *aeruginosa* BSIs fluctuated between 2.37 and 3.51 per 100,000 patient-days over the study period. Out of 285 *P*. *aeruginosa* isolates, 97 (34.04%) were carbapenem-resistant (CR) and 75 (26.32%) were multidrug-resistant (MDR). These isolates showed low resistance to aminoglycosides (9.51–11.62%), broad-spectrum cephalosporins (17.19–17.61%), fluoroquinolones (17.25–19.43%), and polymyxin B (1.69%). The crude 30-day mortality rate was 17.89% (51/285). Healthcare costs of patients with MDR/CR isolates were significantly higher than those of patients with non-MDR/CR isolates (*P* < 0.001/=0.002). Inappropriate definitive therapy [adjusted odds ratio (aOR) 4.47, 95% confidence interval (95% CI) 1.35–14.77; *P* = 0.014], ICU stay (aOR 2.89, 95% CI: 1.26–6.63; *P* = 0.012) and corticosteroids use (aOR 2.89, 95% CI: 1.31–6.41; *P* = 0.009) were independently associated with 30-day mortality.

**Conclusion:**

Incidence of *P*. *aeruginosa* BSIs showed an upward trend during 2017–2020 but dropped in 2021. MDR/CR *P*. *aeruginosa* BSIs are associated with higher healthcare costs. Awareness is required that patients with inappropriate definitive antimicrobial therapy, ICU stay and corticosteroids use are at higher risk of death from *P*. *aeruginosa* BSIs.

## Introduction

Bloodstream infections (BSIs) are common fatal nosocomial infections and pose a significant healthcare issue as they are associated with increased risk of sepsis, hospitalization, healthcare costs and mortality (1, 2). *Pseudomonas aeruginosa* (*P*. *aeruginosa*) accounts for 5.90–15.78% of gram-negative BSIs worldwide (3, 4). A 13-year prospective cohort study conducted in US reported that *P*. *aeruginosa* was responsible for 5.90% of bacterial BSIs (4). BSIs caused by *P*. *aeruginosa* isolates are typically difficult to treat due to remarkable intrinsic antimicrobial resistance and ability to acquire resistance to multiple categories of antimicrobial agents (5). *P*. *aeruginosa* BSIs were associated with increased mortality relative to *Staphylococcus aureus* or other Gram-negative BSIs and this effect persisted after adjustment for patient, bacterial and treatment factors (4). Over the past decade, the overall mortality rates due to *P*. *aeruginosa* BSIs ranged between 1.38 and 37.30% (2, 6–12). These studies have revealed that the epidemiology of *P*. *aeruginosa* BSIs varies geographically.

According to the published data from Blood Bacterial Resistant Investigation Collaborative System in China, *P*. *aeruginosa* was responsible for 5.33% of gram-negative BSIs and the proportion showed an upward trend (13), with a mortality rate ranging from 26.8 to 28.4% (2, 9). There are few published papers on *P*. *aeruginosa* BSIs in China over the past decade (2, 9, 14, 15). Moreover, recent studies addressing risk factors for mortality of *P*. *aeruginosa* BSIs were either small or with focus on multidrug-resistant or carbapenem resistant isolates. To date, no comprehensive epidemiology study of *P*. *aeruginosa* BSIs has been conducted in Hunan Province and Shanghai. As such, we conducted this study with the following aims: (i) to examine incidence of *P*. *aeruginosa* BSIs; (ii) to investigate antimicrobial resistance profile of *P*. *aeruginosa* isolates causing BSIs; (iii) to determine risk factors for all-cause 30-day mortality of *P*. *aeruginosa* BSIs. Findings of this study will shed light on refining local screening and infection control policies for *P*. *aeruginosa* BSIs and thus will prevent further deterioration.

## Methods

### Ethics

The study was reviewed and approved by the participating hospitals (reference number: 202212318, KY2023-083). The need for informed consent was waived due to the observational retrospective nature of the study. The study was conducted in accordance with the Declaration of Helsinki.

### Study design and setting

We performed a retrospective cohort study at two tertiary hospitals between January 1, 2017 and December 31, 2021. Xiangya Hospital is a 3,500-bed hospital located in Changsha, Central China and Ruijin Hospital is a 2,500-bed hospital located in Shanghai, East China. All consecutive hospitalized patients with *P*. *aeruginosa* BSIs admitted during the study period were included. Only the first episode of each patient was included and each patient was included only once. Patients with length of hospitalization < 48 h or incomplete data were excluded.

### Definitions

*P*. *aeruginosa* BSIs were defined as the presence of a positive blood culture of *P*. *aeruginosa* with simultaneous clinical signs and symptoms of infections (16). Onset of *P*. *aeruginosa* BSIs was defined as the moment of taking the first positive blood culture of *P*. *aeruginosa*. A case was defined as a patient diagnosed with *P*. *aeruginosa* BSIs. Incidence of *P*. *aeruginosa* BSIs was defined as the number of cases per 100,000 patient-days. Nosocomial BSIs were defined as blood samples taken more than 48 h after hospital admission and no presence of any clinical signs or symptoms of infections between hospital admission and onset of *P*. *aeruginosa* BSIs (6). Polymicrobial BSIs were defined as recovery of multiple bacterial species from a blood specimen in addition to *P. aeruginosa* (11). Source of infection was defined as the most possible origin of infection responsible for *P. aeruginosa* BSIs according to both medical records and US Centers for Disease Control and Prevention guidelines (9, 17), including an “Unknown” origin if no source was identified. Crude 30-day mortality rate was defined as the number of deaths by any cause within 30 days of onset of *P*. *aeruginosa* BSIs per 100 cases. Inappropriate empirical antimicrobial therapy was defined as no administration of any anti-pseudomonal agent with *in-vitro* activity before blood culture report, whereas inappropriate definitive antimicrobial therapy refers to the moment of receiving antimicrobial susceptibility test results (2, 18).

Both hospitals follow international guideline to collect and process blood cultures (19). Identification of *P. aeruginosa* isolates was performed using MALDITOF MS (bioMérieux, Marcy l'Etoile, France or Zybio Inc., Chongqing, China). Antimicrobial susceptibility test was performed by VITEK^®^2 Compact (bioMérieux, Marcy l'Etoile, France) (20). The sensitivity of polymyxin B was detected using broth microdilution method. The results were interpreted according to the Clinical and Laboratory Standards Institute guidelines (21). Antibiotics tested included amikacin, gentamicin, tobramycin, imipenem, meropenem, ceftazidime, cefepime, ciprofloxacin, levofloxacin, piperacillin/tazobactam, cefoperazone/sulbactam, polymyxin B, and aztreonam. Newer antibiotics such as ceftolozane and ceftazidime/avibactam were not tested as they were either not approved or not widely applied in clinical use during the study period. Multidrug-resistant (MDR) *P*. *aeruginosa* was defined as isolates non-susceptible *in-vitro* to least one agent in three or more antipseudomonal antimicrobial categories (22, 23). Carbapenem-resistant (CR) *P*. *aeruginosa* was defined as isolates non-susceptible *in-vitro* to imipenem or meropenem.

### Data collection

Data were extracted from medical records via the electronic hospital and laboratory information system. All the data were place in one of six categories: (i) age, gender and ward; (ii) comorbidities; (iii) healthcare exposure in the prior 90 days before onset of *P*. *aeruginosa* BSIs including time at risk, intensive care unit (ICU) stay, length of ICU stay and length of hospital stay; (iv) invasive procedures in the prior 90 days before onset of *P*. *aeruginosa* BSIs; (v) drug use 90 days before onset of *P*. *aeruginosa* BSIs until discharge including corticosteroids, immunosuppressor and antibiotics; (vi) date of taking the first positive blood culture of *P*. *aeruginosa*, source of infection and antimicrobial susceptibility results. For risk factor analysis, groups (i)–(v) were considered as potential risk factors. Definition of each variable corresponding to these data was listed in Supplementary Table 1.

### Statistical analysis

The resistance rates were compared between antimicrobial resistant and non-resistant phenotypes using Pearson's Chi-squared test or Fisher's exact test as appropriate. The Kaplan-Meier method was used to plot 30-day survival curves and differences between survival curves by antimicrobial resistant phenotypes were evaluated by the log-rank test (23). A generalized linear mixed model with hospital as a random effect was used to determine risk factors for 30-day mortality and to compare total length of hospital stay and healthcare costs between cases infected with antimicrobial resistant *P*. *aeruginosa* isolates and cases infected with non-resistant *P*. *aeruginosa* isolates. For risk factor analysis, univariate analysis was performed first. Correlation and relevant interactions between variables with *P* < 0.10 in univariate analysis were checked. After removing variables with high-level correlation (correlation coefficient ≥0.70), the remaining variables were considered for inclusion in the multivariate model and selected using lease absolute shrinkage and selection operator (LASSO) penalty (lambda used to choose variables = lambda.1se, the lambda that minimizes cross validation error plus one standard error) (24). The selected variables were included in the final multivariate analysis to determine the independent associations. Odds ratio (OR) and 95% confidence interval (95%CI) were calculated to determine the strengths of these associations. All the analyses were performed using R version 4.2.1 and a *P*-value < 0.05 was considered statistically significant. To test the stability of the final multivariate model, variables in the model were removed in turn and the significance of the remaining variables were checked (25).

## Results

### Overview of the study

A total of 288 cases were diagnosed with *P*. *aeruginosa* BSIs. Two patients with length of hospitalization < 48 h and one patient with incomplete data were excluded, hence only 285 cases were included in the study. The number of cases identified each year was 48, 54, 58, 65 and 60, respectively. Clinical characteristics of *P*. *aeruginosa* BSIs cases are listed in Table 1. The median age of the 285 cases was 55 years and 29.12% were 65 years or older. There were more male cases (190/285, 66.67%). Majority of the 285 cases were nosocomial BSIs (242/285, 84.91%) and 24.56% (70/285) were polymicrobial BSIs. The details of other bacterial species in polymicrobial BSIs cases were listed in Supplementary Table 2. Of the 84 (29.47%) cases with known source, respiratory tract was the most common source (32/285, 11.23%), followed by skin and soft tissue (27/285, 9.47%). Forty-two (14.74%) cases were from ICU, but 30.53% (87/285) had been admitted to ICU in the prior 90 days of *P*. *aeruginosa* BSIs onset. Nearly half (132/285, 46.32%) of all the cases had malignancy. Most of these patients had antimicrobial exposure in the prior 90 days of *P*. *aeruginosa* BSIs onset (240/285, 84.21%).

**Table 1:** Clinical characteristics of 285 *Pseudomonas aeruginosa* bloodstream infections cases.

**Characteristics**	**Number (%)^§^**
Age, years	55 (36–66, 0–97)^¶^
Age ≥ 65 years	83 (29.12)
Gender, male	190 (66.67)
Nosocomial BSIs	242 (84.91)
Polymicrobial BSIs	70 (24.56)
**Ward**	
ICU	42 (14.74)
Medical	121 (42.46)
Surgical	122 (42.81)
**Source of BSIs**	
Unknown	201 (70.53)
Respiratory tract infection	32 (11.23)
Skin and soft tissue infection	27 (9.47)
Gastrointestinal infection	17 (5.96)
Urinary tract infection	6 (2.11)
Catheter-related infection	2 (0.70)
**Comorbidities**	
Agranulocytosis	67 (23.51)
Chemotherapy or radiotherapy	79 (27.72)
Malignancy	132 (46.32)
Disease of the circulatory system	125 (43.86)
Disease of the respiratory system	108 (37.89)
Endocrine, nutritional, and metabolic diseases	89 (31.23)
Diabetes mellitus	36 (12.63)
Chronic renal disease	19 (6.67)
Biliary tract and pancreas diseases	44 (15.44)
Burns	25 (8.77)
**Healthcare exposure**	
Time at risk, days	13 (5–21, 1–334)^¶^
Length of hospital stay, days	18 (9–35, 1–90)^¶^
ICU stay	87 (30.53)
Length of ICU stay, days	0 (0–2, 0–81)^¶^
**Invasive procedures**	
Surgery	132 (46.32)
Invasive ventilation	66 (23.16)
Indwelling catheterization	
CVC	170 (59.65)
Urinary catheter	157 (55.09)
Gastric tube	96 (33.68)
**Drug usage**	
Corticosteroids	164 (57.54)
Immunosuppressor	51 (17.89)
Antibiotics	240 (84.21)

BSIs, bloodstream infections; ICU, intensive care unit; CVC, central venous catheter.

^§^Number of cases with the characteristics, unless stated otherwise.

^¶^Median (interquartile range IQR, range).

### Incidence

**Figure 1:**
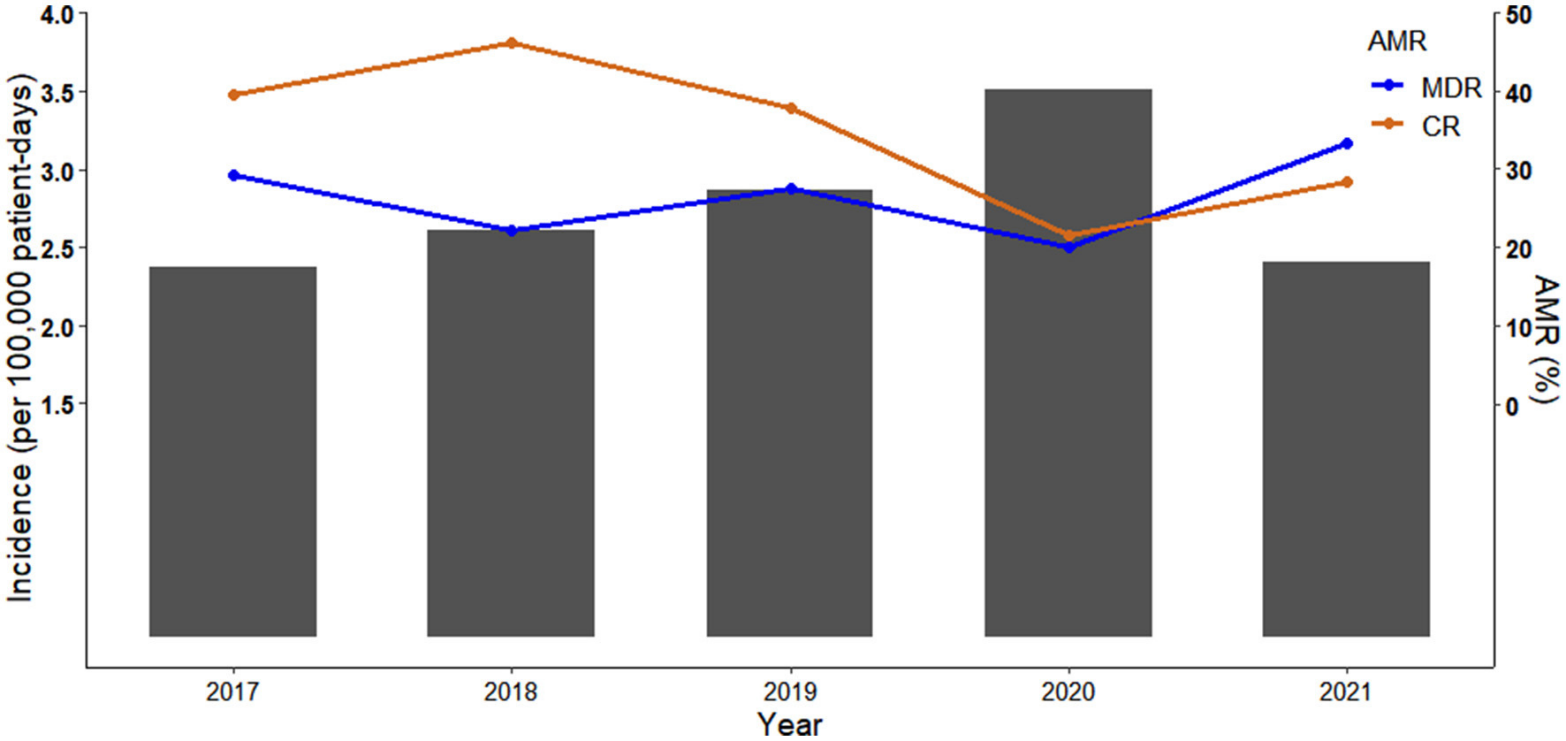
Incidence of *Pseudomonas aeruginosa* bloodstream infections (*P*. *aeruginosa* BSIs) and percentage of antimicrobial resistant phenotypes (AMR) between 2017 and 2021. Bar chart represents the incidence of *P*. *aeruginosa* BSIs. Scatter plot and line chart illustrate percentage of multidrug resistant (MDR) and carbapenem resistant (CR) *P*. *aeruginosa* isolates.

Annual incidence of *P*. *aeruginosa* BSIs fluctuated between 2.37 per 100,000 patient-days and 3.51 per 100,000 patient-days over the study period, showing an upward trend between 2017 and 2020 (Figure 1).

### Antimicrobial resistance

**Figure 2:**
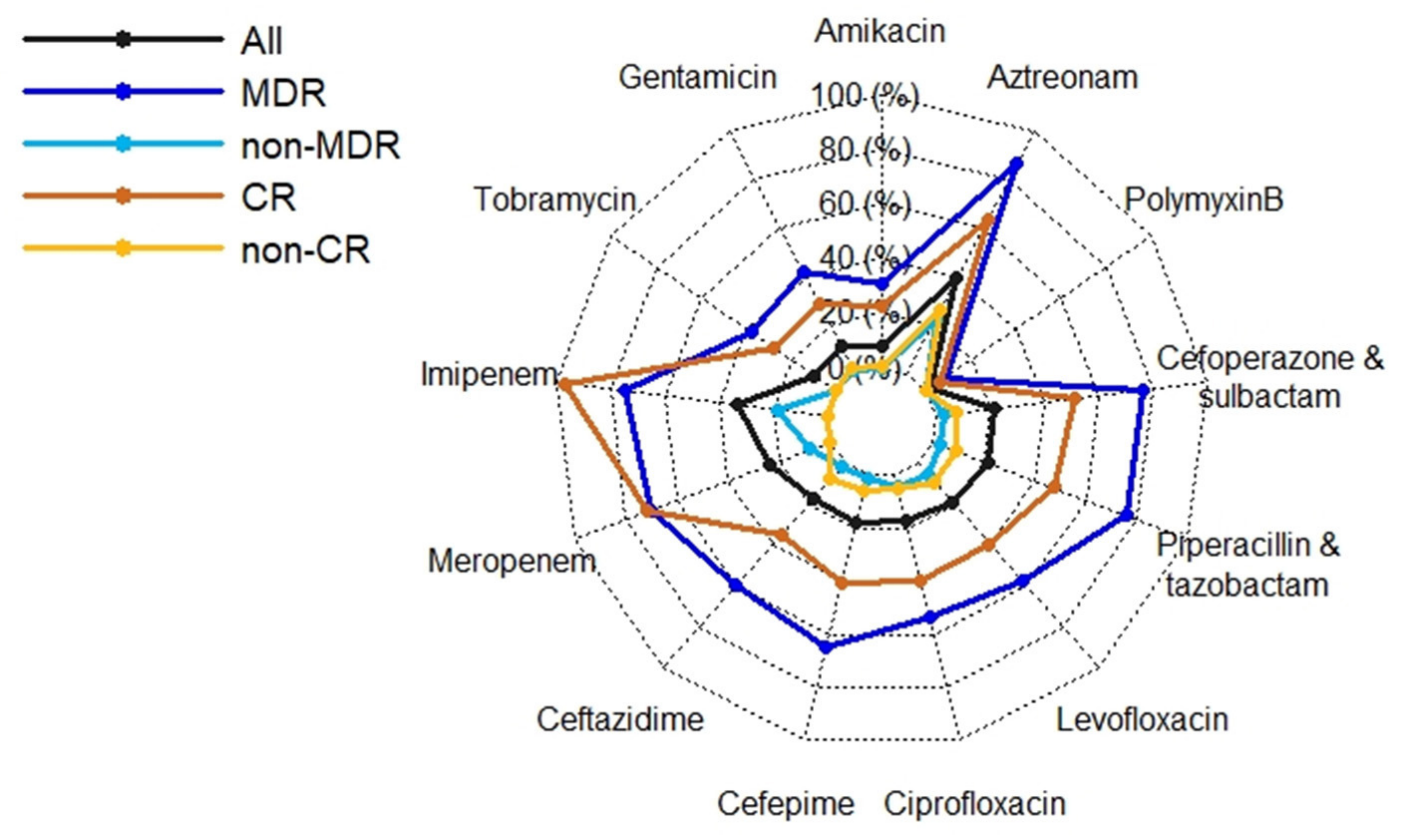
Radar plot of antimicrobial resistance rates of 285 *P*. *aeruginosa* isolates by antimicrobial resistant phenotypes. MDR, multidrug resistant; non-MDR, non-multidrug resistant; CR, carbapenem resistant; non-CR, non-carbapenem resistant.

The MDR and CR phenotypes were present in 26.32% (75/285) and 34.04% (97/285) of the 285 *P*. *aeruginosa* isolates, respectively. The percentage of CR *P*. *aeruginosa* isolates was generally higher than that of MDR isolates in 2017-2020, but there were more MDR isolates than CR isolates in 2021 (Figure 1). All isolates showed low resistance to aminoglycosides (9.51–11.62%), broad-spectrum cephalosporins (17.19–17.61%), fluoroquinolones (17.25–19.43%), and polymyxin B (1.69%) (Figure 2, Supplementary Table 3). By contrast, resistance rate was the highest to aztreonam (39.44%). For all the antibiotics tested, resistance rates of MDR isolates were significantly higher than those of non-MDR isolates (Figure 2, Supplementary Table 3). Also, CR isolates showed significant higher resistance to most antibiotics than non-CR isolates except for polymyxin B (Figure 2, Supplementary Table 3). It is notable that resistance rates of MDR isolates were higher to majority of the antibiotics than those of CR isolates except for carbapenems (Figure 2).

### Mortality and risk factors for crude 30-day mortality

**Figure 3:**
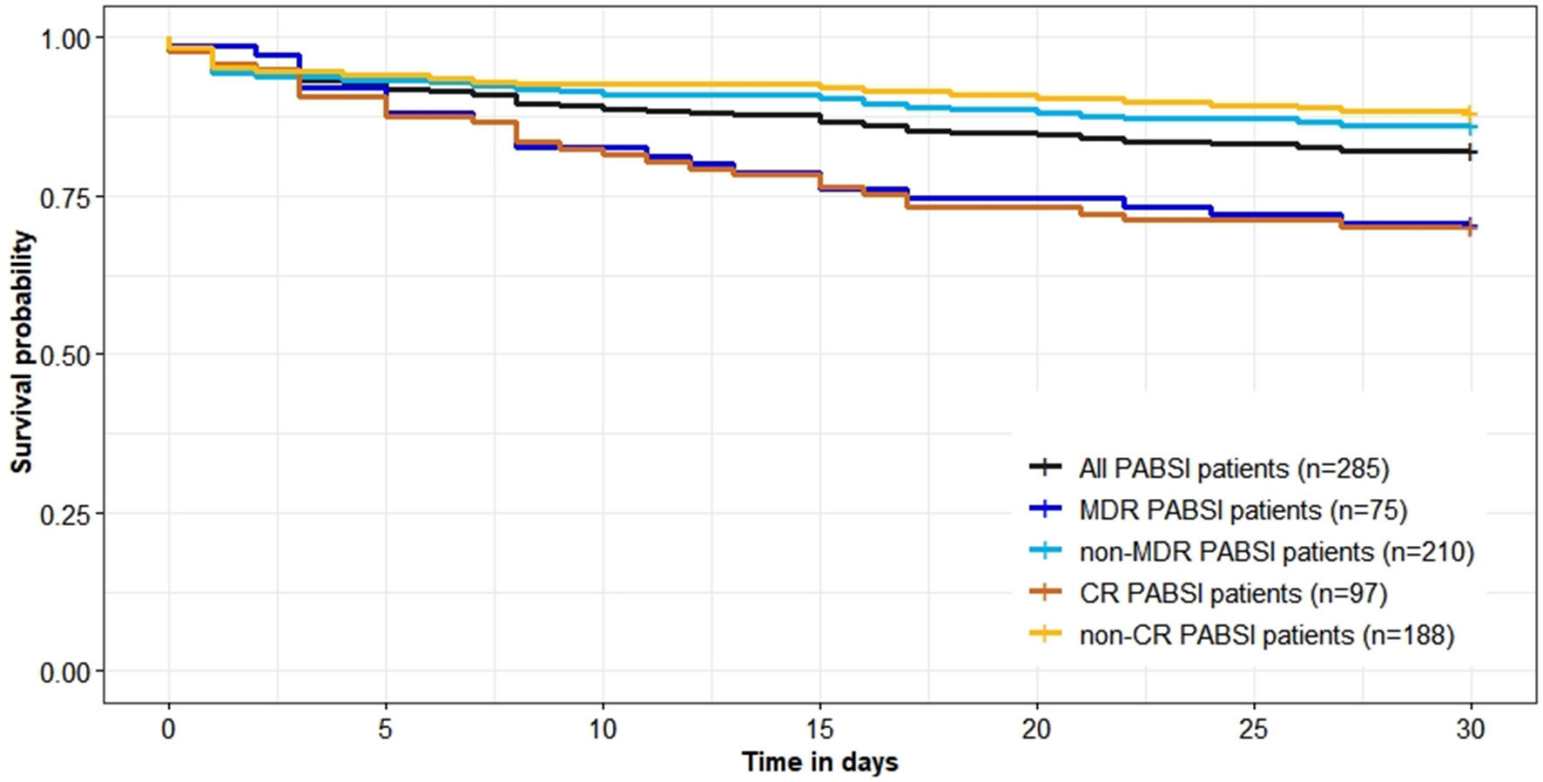
Survival (in days) of 285 *Pseudomonas aeruginosa* bloodstream infections (PABSI) cases and comparison by antimicrobial resistant phenotypes. MDR, multidrug resistant; non-MDR, non-multidrug resistant; CR, carbapenem resistant; non-CR, non-carbapenem resistant.

Crude 30-day mortality rate was 17.89% (51/285). Crude 30-day survival of cases with MDR and CR *P*. *aeruginosa* isolates were significantly lower than those of cases with non-MDR and non-CR isolates (*P* = 0.003/ < 0.001; Figure 3). There were no differences of total length of hospital stay between cases with MDR/CR isolates and cases with non-MDR/non-CR isolates (Table 2). However, healthcare costs of cases with MDR/CR isolates were significantly higher than those of cases with non-MDR/non-CR isolates (Table 2).

**Table 2:** Comparison of total length of hospital stay and healthcare costs of 285 *Pseudomonas aeruginosa* bloodstream infections cases by antimicrobial resistant phenotypes.

**Variable**	**MDR vs. non-MDR**			**CR vs. non-CR**		
	**MDR**	**Non-MDR**	***P***	**CR**	**Non-CR**	***P***
Total length of hospital stay (days), median (IQR, range)	35 (22–77, 6–507)	28 (16–40, 2–467)	0.063	30 (18–53, 5–507)	27 (17–43, 2–467)	0.520
Healthcare costs (10,000 CNY), median (IQR, range)	22.96 (9.13–52.52, 0.62–464.73)	10.65 (5.06–20.37, 0.51–116.27)	< 0.001	18.83 (7.30–43.38, 0.51–464.73)	10.22 (5.05–20.24, 0.62–116.27)	0.002

IQR, interquartile range; CNY, China Yuan; MDR, multidrug resistant; non-MDR, non-multidrug resistant; CR, carbapenem resistant; non-CR, non-carbapenem resistant.

The univariate analysis showed that polymicrobial BSIs, antimicrobial resistant phenotypes (MDR or CR), burns, several healthcare exposure and treatment factors in the prior 90 days before BSIs onset (ICU stay, length of ICU stay, invasive ventilation, indwelling catheterization, corticosteroids, carbapenems, and quantity of carbapenems) and inappropriate empiric and definitive therapies were associated with crude 30-day mortality (Table 3). The multivariate analysis indicated that inappropriate definitive therapy [adjusted odds ratio (aOR) 4.47, 95% confidence interval (95% CI) 1.35–14.77; *P* = 0.014], ICU stay (aOR 2.89, 95% CI: 1.26–6.63; *P* = 0.012) and corticosteroids use (aOR 2.89, 95% CI: 1.31–6.41; *P* = 0.009) in the prior 90 days were independent risk factors for crude 30-day mortality (Table 3).

**Table 3:** Risk factors associated with crude 30-day mortality of 285 *Pseudomonas aeruginosa* bloodstream infections cases.

**Characteristics**	**Non-survivor (%)^§^**	**Survivor (%)^§^**	**Univariate**		**Multivariate**	
	***N*** = **51**	***N*** = **234**	**OR (95% CI)**	***P***	**aOR (95% CI)**	***P***
Age (years), median (IQR)	58 (48–65)	55 (35–66.75)	1.01 (0.99–1.02)	0.446		
Age ≥ 65 years	14 (27.45)	69 (29.49)	0.77 (0.38–1.56)	0.475		
Gender, male	38 (74.51)	152 (64.96)	1.51 (0.76–3.01)	0.242		
Smoking	58 (48–65)	55 (35–66.75)	1.01 (0.99–1.02)	0.446		
Alcohol drinking	14 (27.45)	69 (29.49)	0.77 (0.38–1.56)	0.475		
Polymicrobial BSIs	19 (37.25)	33 (14.10)	3.56 (1.80–7.05)	< 0.001	2.21 (0.98–5.00)	0.056
MDR	22 (43.14)	53 (22.65)	2.41 (1.26–4.61)	0.008		
CR	29 (56.86)	68 (29.06)	3.10 (1.59–6.07)	0.001	1.54 (0.69–3.40)	0.289
**Comorbidities**						
Agranulocytosis	7 (13.73)	60 (25.64)	0.54 (0.22–1.34)	0.184		
Chemotherapy or radiotherapy	9 (17.65)	70 (29.91)	0.56 (0.25–1.25)	0.160		
Malignancy	21 (41.18)	111 (47.44)	0.81 (0.44–1.50)	0.503		
Disease of the circulatory system	27 (52.94)	98 (41.88)	1.60 (0.86–2.94)	0.136		
Hypertension	16 (31.37)	65 (27.78)	1.10 (0.57–2.15)	0.774		
IHD	5 (9.80)	16 (6.84)	1.58 (0.54–4.57)	0.403		
Disease of the respiratory system	14 (27.45)	75 (32.05)	0.93 (0.46–1.89)	0.846		
Endocrine, nutritional, and metabolic diseases	7 (13.73)	29 (12.39)	1.21 (0.49–2.97)	0.678		
Diabetes mellitus	20 (39.22)	88 (37.61)	1.59 (0.76–3.31)	0.217		
Chronic renal disease	6 (11.76)	13 (5.56)	2.37 (0.84–6.67)	0.101		
Biliary tract and pancreas diseases	5 (9.80)	39 (16.67)	0.55 (0.20–1.48)	0.234		
Burns	10 (19.61)	15 (6.41)	3.17 (1.3–7.78)	0.012		
**Healthcare exposure**						
Time at risk (days), median (IQR)	14 (8–26.5)	13 (5–21)	1.00 (0.99–1.01)	0.983		
Length of hospital stay (days), median (IQR)	20 (9–28.50)	17.50 (9–38)	0.99 (0.98–1.01)	0.393		
ICU stay	27 (52.94)	60 (25.64)	3.93 (2.03–7.58)	< 0.001	2.89 (1.26–6.63)	0.012
Length of ICU stay (days), median (IQR)	1 (0–11)	0 (0–1)	1.02 (1.00–1.04)	0.042		
**Invasive procedures**						
Surgery	29 (56.86)	103 (44.02)	1.47 (0.77–2.83)	0.243		
Invasive ventilation	21 (41.18)	45 (19.23)	2.95 (1.54–5.66)	0.001		
Indwelling catheterization						
CVC	36 (70.59)	134 (57.26)	2.02 (1.03–3.96)	0.040		
Urinary catheter	41 (80.39)	116 (49.57)	4.06 (1.87–8.79)	< 0.001	1.66 (0.63–4.39)	0.308
Gastric tube	25 (49.02)	71 (30.34)	2.11 (1.14–3.94)	0.018		
**Drug use**						
Corticosteroids	40 (78.43)	124 (52.99)	3.67 (1.77–7.63)	< 0.001	2.89 (1.31–6.41)	0.009
Immunosuppressor	9 (17.65)	42 (17.95)	1.11 (0.49–2.51)	0.796		
Antibiotics	46 (90.2)	194 (82.91)	1.69 (0.62–4.58)	0.304		
Total quantity (DDD), median (IQR)	19.50 (5.50–38.62)	11.13 (2.58–29.05)	1.00 (1.00–1.01)	0.330		
Aminoglycosides	3 (5.88)	12 (5.13)	0.98 (0.26–3.68)	0.978		
Quantity (DDD), median (IQR)	0 (0–0)	0 (0–0)	0.95 (0.80–1.14)	0.584		
Carbapenems	35 (68.63)	82 (35.04)	3.88 (2.00–7.50)	< 0.001	1.71 (0.79–3.73)	0.177
Quantity (DDD), median (IQR)	6 (0–10.50)	0 (0–4.46)	1.03 (1.00–1.06)	0.033		
Broad-spectrum cephalosporins	14 (27.45)	52 (22.22)	1.17 (0.58–2.38)	0.663		
Quantity (DDD), median (IQR)	0 (0–0.88)	0 (0–0)	1.01 (0.97–1.06)	0.555		
β-lactam/β-lactamase inhibitor combinations	10 (19.61)	77 (32.91)	0.51 (0.25–1.29)	0.180		
Quantity (DDD), median (IQR)	0 (0–0)	0 (0–2.97)	0.98 (0.93–1.03)	0.346		
Fluoroquinolones	12 (23.53)	44 (18.8)	1.41 (0.68–2.94)	0.360		
Quantity (DDD), median (IQR)	0 (0–0)	0 (0–0)	1.01 (0.95–1.08)	0.695		
**Antimicrobial therapy after BSIs onset**						
Inappropriate empiric therapy	13 (25.49)	28 (11.97)	2.48 (1.17–5.24)	0.018		
Inappropriate definitive therapy	9 (17.65)	10 (4.27)	4.96 (1.87–13.16)	0.001	4.47 (1.35–14.77)	0.014

OR (95% CI), odds ratio (95% confidence interval); aOR (95% CI), adjusted odds ratio (95% confidence interval); IQR, interquartile range; BSIs, bloodstream infections; MDR, multidrug resistant; non-MDR, non-multidrug resistant; CR, carbapenem resistant; non-CR, non-carbapenem resistant; IHD, ischemic heart disease; ICU, intensive care unit; CVC, central venous catheter; DDD, defined daily dose.

^§^Number of non-survivors/survivors with the characteristics (percentage of non-survivors/survivors with the characteristics), unless stated otherwise.

## Discussion

This study conducted an in-depth epidemiological analysis of *P*. *aeruginosa* BSIs at individual level hence to provide a comprehensive understanding of characteristics underlying factors and related morbidity and mortality associated with *P*. *aeruginosa* BSIs. To the authors' knowledge, this is the first epidemiological study of *P*. *aeruginosa* BSIs conducted in these two different administrative regions of China.

Incidence of *P*. *aeruginosa* BSIs fluctuated between 2.37 and 3.51 per 100,000 patient-days. This finding is similar to a previous study conducted in Southeast China reporting that the incidence was between 2.70 and 6.20 per 100,000 patient-days (2). According to the management policy of coronavirus disease 2019 (COVID-19) over the study period, all COVID-19 patients were closed loop transferred to designated hospitals immediately after positive PCR test at admission. Therefore, none of the 285 *P*. *aeruginosa* BSIs were COVID-19 patients. Also, it is difficult to evaluate impact of the pandemic on *P*. *aeruginosa* BSIs. Incidence of *P*. *aeruginosa* BSIs showed an upward trend during the pre-pandemic (2017–2019) and pandemic period (2020) but dropped in 2021. Some studies observed a higher incidence of BSIs during the COVID-19 pandemic (26–28). As management of COVID-19 has been downgraded in China, impact of the COVID-19 pandemic on the incidence of *P*. *aeruginosa* BSIs warrants more local research in future study. Crude 30-day mortality rate (17.89%) of *P*. *aeruginosa* BSIs was lower than that of studies conducted in other parts of China (2, 9), Europe (6, 10, 29), and Australia (12).

As other studies reported, there were more male cases than female cases (8, 9, 29, 30), however, gender is not associated with adverse clinical outcomes. Yoon et al. have claimed that 26.70% of *P*. *aeruginosa* BSIs patients were with polymicrobial BSIs and this was not associated with mortality though (11). The rate of polymicrobial BSIs in this study was slightly lower (24.56%) than the above study, however, polymicrobial BSIs was a risk factor for crude 30-day mortality. Our data showed that the percentage of ICU stay among cases with polymicrobial BSIs was higher than that among cases only with *P*. *aeruginosa* BSIs (42.31 vs. 27.90%), indicating that polymicrobial BSIs may be a surrogate marker of critical illness and higher risk of death. Respiratory tract (32/84, 38.10%) was the most common probable source among cases with known source of BSIs which agreed with other studies (2, 29, 30). Interestingly, more than half of the 32 cases (20/32, 62.50%) with BSIs stemmed from respiratory tract infection had invasive ventilation support. The mucosal barrier injury of respiratory tract would decrease the capacity for bacterial clearance and increase probabilities of bacterial colonization and/or infection (31).

Almost all of comorbidities were not linked to mortality with the exception of burns (Table 3). Burned patients are more likely to have invasive treatments and are more debilitated and prone to subsequent infections as burn wounds are favorable sites for bacterial colonization until they are closed (32). Our study identified that mechanical ventilation, CVC, urinary catheter and gastric tube were predictors for crude 30-day mortality (Table 3). Invasive indwelling devices or procedures have been widely investigated as risk factors for mortality caused by bacterial infections as bypass the innate host mechanical defenses and provide a niche for microorganisms, facilitating progression of infections (29, 30). Mechanical ventilation is a treatment option that is often necessary in critical ill patients (ICU patients in particular). All the above factors should be interpreted with caution as they may present surrogate markers of critical illness and extensive healthcare exposure rather than reflecting a direct association. Consistent with this interpretation, our study also identified that ICU stay was independently associated with crude 30-day mortality which has been reported by multiple other studies (8, 11, 29). Nevertheless, the exist of *P. aeruginosa* isolates in the manmade environment is a prerequisite for the infection to occur, so implementation and good compliance of aseptic technique during invasive procedures and infection control measures during daily medical work.

In contrast to another study conducted in China (33), our study showed that *P. aeruginosa* isolates retained susceptibility to aminoglycosides, broad-spectrum cephalosporins, fluoroquinolones, and polymyxin B which was consistent with the findings of studies conducted in Spain and Korea (10, 34). Discrepancies between studies may reflect different antimicrobial prescribing practices and antimicrobial mechanisms, highlighting the importance of understanding local anti-biograms to preserve antibiotic utility and rational treatment. MDR and CR phenotypes were widely reported as predictors of poorer outcome as very few effective therapeutic options are available to treatment (2, 6, 9, 10, 29). Although antimicrobial phenotypes were not independently associated with mortality in this study (Table 3) which agrees with Montero et al. (35), they had significant adverse impact on the crude 30-day survival (Figure 3).

We found that prior carbapenems use, quantity of carbapenems, and inappropriate empirical/definitive antimicrobial therapy were linked to mortality (Table 3). Again, this could be explained by critical illness as carbapenems are often used as last resort antibiotics for treatments of MDR infections. It has been well-proved that inappropriate empirical and/or definitive antimicrobial therapy was associated with increased mortality outcomes (2, 8, 10, 30). Moreover, another concern is the higher percentage of MDR isolates over that of CR isolates in 2021 (Figure 1) given that resistance rates of MDR isolates were higher to majority of the antibiotics than those of CR isolates (Figure 2, Supplementary Table 3). These findings would trigger antimicrobial stewardship programs to monitor antimicrobial use and contain antimicrobial resistance in patients with *P*. *aeruginosa* BSIs. In addition, we identified corticosteroids administration as an independent predictor of crude 30-day mortality (Table 3). Corticosteroids are well-recognized as the marker of immunocompromised status which is vulnerable to severe infections. Therefore, past work has widely demonstrated an association between corticosteroids administration and higher mortality risk (2, 8, 12, 29).

Interestingly, we found that total length of hospital stay did not differ significantly between cases with MDR/CR phenotypes and cases with non-MDR/CR phenotypes while healthcare costs of cases with MDR/CR phenotypes were significantly higher than those of cases with non-MDR/non-CR phenotypes (Table 2). Increased healthcare costs may stem from higher costs of agents needed to treat MDR/CR BSIs, greater likelihood for procedures such as line placement for intravenous antibiotics, and complications from these agents and procedures (36–38).

There are some limitations of the study. First, for antimicrobial susceptibility test, not all the isolates were tested for the same agents. Second, no genomic data were available to identify mechanisms of antimicrobial resistance, possible clonal spread and virulence. Bioinformatic and phylogenetic study is warranted to better understand of the phylogeny and pathogenicity of these *P. aeruginosa* isolates in the future.

## Conclusion

In conclusion, annual incidence of *P*. *aeruginosa* BSIs was fluctuating over the study period. *P*. *aeruginosa* BSIs cases with non-MDR/CR phenotypes had a survival advantage over cases with MDR/CR phenotypes which resulted in higher healthcare costs. Awareness is required that patients with inappropriate definitive therapy, ICU stay and corticosteroids use are at higher risk of death from *P*. *aeruginosa* BSIs. *P*. *aeruginosa* isolates retained susceptibility to aminoglycosides, broad-spectrum cephalosporins, fluoroquinolones, and polymyxin B which may be alternative therapeutic options.

## Data availability statement

The original contributions presented in the study are included in the article/Supplementary material, further inquiries can be directed to the corresponding author.

## Ethics statement

The studies involving humans were approved by Medical Ethics Committee of Xiangya Hospital Central South University and Ruijin Hospital Ethics Committee (Shanghai Jiao Tong University School of Medicine). The studies were conducted in accordance with the local legislation and institutional requirements. The human samples used in this study were acquired from a by- product of routine care or industry. Written informed consent for participation was not required from the participants or the participants' legal guardians/next of kin in accordance with the national legislation and institutional requirements.

## Author contributions

SX: Data curation, Formal analysis, Investigation, Methodology, Resources, Software, Validation, Writing—original draft. XL: Data curation, Formal analysis, Investigation, Methodology, Resources, Software, Validation, Writing—original draft. LH: Resources, Validation, Writing—review & editing. SZ: Conceptualization, Formal analysis, Methodology, Project administration, Resources, Supervision, Validation, Writing—review & editing.
