# Supplementary material for: Incidence, antimicrobial resistance and mortality of Pseudomonas aeruginosa bloodstream infections among hospitalized patients in China: a retrospective observational multicenter cohort study from 2017 to 2021
Source: Front Public Health. 2024 Jan 5;11:1294141. doi: 10.3389/fpubh.2023.1294141 (PMC10797092; doi:10.3389/fpubh.2023.1294141)
Supplement: Supplementary file 1 [file Table_1.DOCX]

**Supplementary table 1. Definitions of each independent variable included in risk factors analysis for crude 30-day mortality and clinical characteristics of 285 *Pseudomonas aeruginosa* bloodstream infections cases**

| Variables | Definition |
| --- | --- |
| Age, years | Age at *Pseudomonas aeruginosa* bloodstream infections diagnosis |
| Age≥65 years | Age≥65 years old at *Pseudomonas aeruginosa* bloodstream infections diagnosis |
| Gender, male | As described in section “Gender” of the medical records |
| Smoking | As described in section “Smoking” of the medical records |
| Alcohol drinking | As described in section “Alcohol use” of the medical records |
| Comorbidities | *According to International Classification of Diseases-10th Revision codes (ICD-10) [1]* |
| Agranulocytosis | D70 |
| Chemotherapy or radiotherapy | Receiving chemotherapy or radiotherapy treatment as described in medical records |
| Malignancy | C00-C80, C7A, C81-C96, D00-D09 |
| Disease of the circulatory system | I00-I99 |
| Hypertension | I10-I16 |
| IHD | I25 |
| Disease of the respiratory system | J00-J99 |
| Endocrine, nutritional and metabolic diseases | E00-E89 |
| Diabetes mellitus | Including Type 1 and Type 2 |
| Chronic renal disease | N18 |
| Biliary tract and pancreas diseases | K80-K81, K85-K86 |
| Burns | T20-T28, T30-T32 |
| Healthcare exposure | *In the prior 90 days before blood collection date of the first positive culture, unless stated otherwise.* |
| Time at risk, days | For the index hospitalization, interval between hospital admission and blood sample collection date of the first positive culture |
| Length of hospital stay, days | Total days of hospital stay |
| ICU stay | Stayed in ICU |
| Length of ICU stay, days | Total days of ICU stay |
| Invasive procedures | *In the prior 90 days before blood collection date of the first positive culture* |
| Surgery | As described in section “Surgery” of the medical records |
| Invasive ventilation | Invasive ventilation |
| Indwelling catheterization | Insertion of indwelling devices, including CVC, urinary catheter, gastric tube, dialysis or drainage requiring indwelling catheterisation, cannulation of artery |
| CVC | Insertion of CVC |
| Urinary catheter | Insertion of urinary catheter |
| Gastric tube | Insertion of gastric tube |
| Drug usage | *Any drug treatment in the prior 90 days before blood collection date of the first positive culture, including corticosteroids, immunosuppressor and antibiotics* |
| Corticosteroids | Administration of corticosteroids |
| Immunosuppressor | Administration of immunosuppressor, including cyclophosphamide, methotrexate, cyclosporine, tacrolimus |
| Antibiotics | Any antibiotics usage for at least 72 hours |
| Total quantity, DDD | Total quantity of all the antibiotics used |
| Aminoglycosides | Administration of any aminoglycoside |
| Quantity, DDD | Total quantity of all the aminoglycosides used |
| Carbapenems | Administration of any carbapenem |
| Quantity, DDD | Total quantity of all the carbapenems used |
| Broad-spectrum cephalosporins | Administration of any third- or fourth-generation cephalosporin |
| Quantity, DDD | Total quantity of all the broad-spectrum cephalosporins used |
| β-lactam/β-lactamase inhibitor combinations | Administration of any β-lactam/β-lactamase inhibitor combination |
| Quantity, DDD | Total quantity of all the β-lactam/β-lactamase inhibitor combinations used |
| Fluoroquinolones | Administration of any fluoroquinolone |
| Quantity, DDD | Total quantity of all the fluoroquinolones used |

IHD, ischemic heart disease; ICU, intensive care unit; CVC, central venous catheter; DDD, defined daily dose.

References:

[1] Centers for Disease Control and Prevention. *International Classification of Diseases-10th Revision codes*. 2017. Available from: <https://www.cdc.gov/nchs/icd/icd-10-cm.htm> (last accessed March 2023)

**Supplementary table 2. Other bacterial species detected with *Pseudomonas aeruginosa* of 70 polymicrobial bloodstream infections cases**

| **Genus** | **Species** | **Number** |
| --- | --- | --- |
| *Klebsiella* | *Klebsiella pneumonia* | 13 |
| *Escherichia* | *Escherichia coli* | 10 |
| *Serratia* | *Serratia marcescens* | 2 |
| *Enterobacter* | *Enterobacter cloacae* | 1 |
|  | *Enterobacter aerogenes* | 1 |
| *Acinetobacter* | *Acinetobacter baumannii* | 4 |
|  | *Acinetobacter pittii* | 1 |
| *Pseudomonas* | *Pseudomonas putida* | 2 |
| *Ralstonia* | *Ralstonia mannitolilytica* | 1 |
| *Aeromonas* | *Aeromonas caviae* | 1 |
| *Enterococcus* | *Enterococcus faecium* | 4 |
|  | *Enterococcus faecalis* | 1 |
| *Staphylococcus* | *Staphylococcus aureus* | 5 |
|  | *Staphylococcus haemolyticus* | 3 |
|  | *Staphylococcus capitis* | 2 |
|  | *Staphylococcus hominis* | 1 |
|  | *Staphylococcus warneri* | 1 |
| *Streptococcus* | *Streptococcus pneumoniae* | 1 |
|  | *Streptococcus oralis* | 1 |
| *Candida* | *Candida tropical* | 2 |
|  | *Candida albicans* | 1 |
|  | *Candida parapsilosis* | 1 |
| Mixed | *Acinetobacter baumannii+Klebsiella pneumonia+Stenotrophomonas maltophilia* | 1 |
|  | *Klebsiella pneumonia+Stenotrophomonas maltophilia+Candida tropical* | 1 |
|  | *Acinetobacter baumannii+Klebsiella pneumonia* | 1 |
|  | *Acinetobacter baumannii+Stenotrophomonas maltophilia* | 1 |
|  | *Acinetobacter baumannii+Candida tropical* | 1 |
|  | *Klebsiella oxytoca*+*Enterococcus faecalis* | 1 |
|  | *Enterobacter cloacae+Stenotrophomonas maltophilia* | 1 |
|  | *Klebsiella pneumonia+Staphylococcus capitis* | 1 |
|  | *Candida glabrata +Acinetobacter pittii* | 1 |
|  | *Staphylococcus haemolyticus+Streptococcus mitis* | 1 |
|  | *Candida parapsilosis+Staphylococcus hominis* | 1 |

**Supplementary table 3. Antimicrobial resistance rates of *Pseudomonas aeruginosa* isolates and comparison by antimicrobial resistant phenotypes**

| **Agents** | **All isolates** | **MDR^§^** | | | **non-MDR^§^** | ***P**** | **CR^§^** | **non-CR^§^** | ***P**** |
| --- | --- | --- | --- | --- | --- | --- | --- | --- | --- |
| Aminoglycoside |  |  | | |  |  |  |  |  |
| Amikacin | 23/284 (9.51) | 23/75 (30.67) | | | 0/209 (0.00) | <0.001 | 22/97 (22.68) | 1/187 (0.53) | <0.001 |
| Gentamicin | 28/241 (11.62) | 26/62 (41.94) | | | 2/179 (1.12) | <0.001 | 24/83 (28.92) | 4/158 (2.53) | <0.001 |
| Tobramycin | 28/283 (9.89) | 28/74 (37.84) | | | 0/209 (0.00) | <0.001 | 27/96 (28.13) | 1/187 (0.53) | <0.001 |
| Carbapenem |  |  | | |  |  |  |  |  |
| Imipenem | 94/284 (33.10) | 56/75 (74.67) | | | 38/209 (18.18) | <0.001 | 94/97 (96.91) | 0/187 (0.00) | <0.001 |
| Meropenem | 66/275 (24.00) | 50/71 (70.42) | | | 16/204 (7.84) | <0.001 | 66/92 (71.74) | 0/183 (0.00) | <0.001 |
| Cephalosporin |  |  | | |  |  |  |  |  |
| Ceftazidime | 49/285 (17.19) | 45/75(60.00) | | | 4/210 (1.90) | <0.001 | 34/97 (35.05) | 15/188 (7.98) | <0.001 |
| Cefepime | 50/284 (17.61) | 48/74 (64.86) | | | 2/210 (0.95) | <0.001 | 39/96 (40.63) | 11/188 (5.85) | <0.001 |
| Fluoroquinolone |  |  | | |  |  |  |  |  |
| Ciprofloxacin | 49/284 (17.25) | 40/75 (53.33) | | | 9/209 (4.31) | <0.001 | 39/97 (40.21) | 10/187 (5.35) | <0.001 |
| Levofloxacin | 55/283 (19.43) | 43/74 (58.11) | | | 12/209 (5.74) | <0.001 | 38/96 (39.58) | 17/187 (9.09) | <0.001 |
| β-Lactam/β-lactamase inhibitor combinations | | |  |  |  |  |  |  |  |
| Piperacillin/tazobactam | 63/285 (22.11) | 57/75 (76.00) | | | 6/210 (2.86) | <0.001 | 46/97 (47.42) | 17/188 (9.04) | <0.001 |
| Cefoperazone/sulbactam | 58/267 (21.72) | 52/68 (76.47) | | | 6/199 (3.02) | <0.001 | 44/86 (51.16) | 14/181 (7.73) | <0.001 |
| PolymyxinB | 2/118 (1.69) | 2/23 (8.70) | | | 0/95 (0.00) | 0.0367^¶^ | 2/32 (6.25) | 0/86 (0.00) | 0.0719^¶^ |
| Aztreonam | 112/284 (39.44) | 65/75 (86.67) | | | 47/209 (22.49) | <0.001 | 62/97 (63.92) | 50/187 (26.74) | <0.001 |

MDR, multidrug resistant; non-MDR, non-multidrug resistant; CR, carbapenem resistant; non-CR, non-carbapenem resistant.

^§^, Number of resistant isolates/number of isolates tested (percentage of resistance).

*, Pearson’s Chi-squared test, unless stated otherwise.

^¶^, Fisher’s exact test.
